# Patient reflections on benefits, challenges, and improvement opportunities for a VA podiatry telehealth program: a qualitative study

**DOI:** 10.3389/fdgth.2026.1904512

**Published:** 2026-08-03

**Authors:** Lacey Evans, Tammy L. Eaton, Kennedy L. Cioccio, Jessica L. Johnson, C. Ann Vitous, Kaylee W. Burgan, John P. Donnelly, Nicholas W. Bowersox, Nichol L. Salvo, Sherill Murad, Ashley Willis, Linda M. Kawentel

**Affiliations:** 1 VA QUERI Center for Evaluation and Implementation Resources, Ann Arbor, MI, United States; 2 Department of Internal Medicine, Division of Hospital Medicine, Michigan Medicine, University of Michigan, Ann Arbor, MI, United States; 3 VA Center for Clinical Management Research, Ann Arbor, MI, United States; 4 Department of Learning Health Sciences, University of Michigan, Ann Arbor, MI, United States; 5 Department of Psychiatry, University of Michigan, Ann Arbor, MI, United States; 6 Atlanta VA Health Care System, Atlanta, GA, United States

**Keywords:** patient interviews, podiatry, qualitative, telehealth, telemedicine

## Abstract

**Introduction:**

Within the Department of Veterans Affairs (VA), the High-risk Eye and Limb Preservation Program (HELPP) delivers basic foot care using telemedicine and remote technology to prevent Veteran limb amputation. To enable basic foot care delivery in rural locations, HELPP hires intermediate care technicians (ICTs) to perform foot care under the tele-supervision of a podiatrist. This evaluation aimed to understand HELPP benefits, challenges, and improvement opportunities based on patients’ experiences.

**Methods:**

As part of a program evaluation, patients who had received HELPP care were invited to participate in semi-structured interviews informed by the RE-AIM framework to assess patient-experienced benefits and challenges with HELPP and identify improvement opportunities. Rapid analysis was performed using summary templates and an analytic matrix.

**Results:**

Twenty-one interviews were completed. Benefits included staff quality, increased convenience and accessibility, and improved and maintained foot health. Challenges included ICT turnover and the resulting issues with variability in communication and care quality, the podiatrist not being in-person, and scheduling issues. Patients’ improvement suggestions focused on connecting more Veterans to care and follow-up care.

**Discussion:**

Overall, the patients interviewed were satisfied with their HELPP care and identified several benefits; however, patients also spoke to challenges and improvement opportunities that should be considered for HELPP expansion and sustainability. Notably, patients often spoke of staff quality and effect of staff turnover, highlighting the importance and visibility of staffing in telemedicine care.

## Introduction

Telemedicine has become more widespread following the COVID-19 pandemic (1, 2), improving access to care, particularly for patients in rural settings (2). Despite these gains, uncertainty remains regarding which types of care are well-suited for telemedicine delivery (1). Concerns persist about limitations in conducting physical examinations remotely, highlighting the need for care models that balance access with care quality (3). Hybrid telehealth models, in which patients alternate between in-person and virtual visits, represent one approach; however, these models often still rely on patient access to technology and digital literacy (4).

Within the Department of Veterans Affairs (VA), intermediate care technicians (ICTs), often former military medics and corpsmen, are employed to deliver in-person physical exams while clinicians provide care via tele-supervision (5, 6). This model represents a hybrid workforce innovation that extends reach of healthcare services through task-sharing while removing the burden of technology use from patients. Although this workforce innovation shows promise, patient perspectives on ICT-mediated hybrid telehealth models remain underexplored.

In 2020, VA launched the High-risk Eye and Limb Prevention Program (HELPP) to improve access to foot care for patients, particularly patients with diabetes, with the goal of reducing complications such as limb amputations (7). HELPP utilizes ICTs to perform foot exams and care under synchronous podiatrist tele-supervision at community-based outpatient clinics (CBOCs) that otherwise lack podiatry services.

To assess whether HELPP is meeting patient needs, this evaluation examined patient experiences with HELPP. This evaluation explored patient-reported benefits, challenges, and opportunities for improvement to inform program refinement and broader implementation considerations.

## Methods

### Ethics approval

This program evaluation qualified as a non-research, as it was designed for internal quality improvement efforts, as described in the VHA Program Guide 1200.21, and thus did not require IRB review (8). The evaluation was conducted under the authority of the VHA Specialty Care Program Office.

### Study design and sample

As part of the evaluation of HELPP, the evaluation team and HELPP clinical team collaboratively created a semi-structured interview guide to assess patient-perceived benefits, challenges, and opportunities for improvement. The guide was informed by the RE-AIM framework (9, 10), and revised after review by a Veterans engagement council that provides feedback on project procedures and materials. The final interview guide is included as Supplementary File 1.

Patients within one healthcare system were purposively sampled based on participation in at least one HELPP visit, access to a phone or computer with reliable internet connection, and the ability to consent and participate in an interview. The target sample size of 20 participants was determined before data collection to balance the need for diverse perspectives with available evaluation resources and timelines. Because participants shared a common experience with the program, the target sample size was expected to provide sufficient informational power to identify key experiences relevant to the evaluation objectives (11). Thirty-one patients were recruited by the HELPP clinical team (NLS, SM, and AW), who provided an overview of the evaluation and obtained patients’ written consent to release contact information to the evaluation team. The evaluation project coordinator (KLC) then called or emailed each of the 31 patients to schedule them for an interview. At the beginning of each interview, the interviewer (LE, TLE, CAV, KWB, LMK) obtained verbal consent to conduct and record the interview.

### Data collection

Interviews were conducted by a trained interviewer (LE, TLE, CAV, KWB, LMK) with a notetaker present (LE, KLC, JLJ, CAV, KWB, LMK) between May and August 2025. Interviews averaged 35 minutes in length (range: 20–60 minutes). All interviews were audio-recorded and transcribed verbatim. Confidentiality was maintained by storing consent forms, recordings, and transcripts behind a secure server only accessible to evaluation staff. Prior to sharing results, transcripts were reviewed for accuracy and completeness, personal references were removed, and quotations were attributed to unique participant IDs rather than by name.

### Data analysis

Rapid qualitative analysis was completed by three trained analysts (LE, TLE, LMK) guided by Hamilton's approach of using summary templates and a matrix to facilitate analysis (12). Each interview was assigned to primary and secondary analysts. The primary analyst completed the summary template in Microsoft Word and a second analyst reviewed and refined content for completeness and accuracy.

Summaries were then used to populate an analytic matrix in Microsoft Excel with domains derived from interview questions. Focused coding using inductive codes was then applied to each domain column within the matrix. In parallel, analysts produced concise summaries for each domain. Consensus discussion between all three analysts occurred regularly. When disagreements occurred between the primary and secondary analysts, the third analyst weighed in to make a final decision.

## Results

Twenty-one semi-structured interviews were completed (response rate: 68%) with patients receiving HELPP care across four clinics within one VA healthcare system (Table 1). Patient reflections on HELPP were organized into three domains: 1) benefits, 2) challenges, and 3) suggestions for improvement.

**Table 1:** Key veteran participant characteristics (*N* = 21 veterans).

Participant characteristics	n	%
Primary Location		
VAMC	2	9.5%
CBOC A	4	19.1%
CBOC B	8	38.1%
CBOC C	7	33.3%
Patient-identified diabetes diagnosis	12	57.1%
Time in VA Podiatry Care		
Less than a year	1	4.8%
1–3 years	7	33.3%
4–5 years	4	19.1%
Over 5 years	8	38.1%
Unknown	1	4.8%
HELPP Referral Source		
Primary Care	13	62.0%
Specialty Care	1	4.8%
Not Known	7	33.3%
Experience with Community Care		
Yes	6	28.6%
No	15	71.4%

VAMC, VA medical center; CBOC, community-based outpatient clinic.

### Benefits

When asked about the best part of their HELPP care, patients most frequently focused on staff quality, describing HELPP providers and staff as professional, welcoming, and capable.

‘The technicians are conscientious, they are meticulous, and they are professional […] the podiatrist is always authentically caring, patient, and extremely knowledgeable. And the technicians, they provide splendid foot care.’ (Patient 17)

Convenience was a key benefit, particularly reduced travel time and avoidance of traffic and parking challenges due to receiving care at a CBOC, which are smaller than VA medical centers and may be closer to rural patients’ homes.

‘So, the ability to get to the [CBOC] location greatly improves my ability to make appointments. Because to get to [CBOC] is only about a total 20, 25-minute drive from where I live. That's a big plus.’ (Patient 5)

Additional benefits included short wait times and accessible appointments, continuity of care, frequency of visits, and the ability to get new shoes and socks through the program.

‘Well, I can look forward to every three months, my feet [are] going to feel good […] Every year I'm gonna have a new pair of shoes that I could pick my—the styles that they have, I can pick that. I don't have to take what they give me. They have a variety that you can pick from.’ (Patient 20)

Importantly, patients spoke of the improved and maintained foot health they experienced due to their HELPP care.

‘The increase in my mobility is the best part. That's what overrides everything else in my mind because I was always an active person. And when I became, shall I say, house-active only, I was not a happy camper. That I have this ability now to go out whenever I want to I consider the biggest plus from this entire experience.’ (Patient 5)

### Challenges

Despite overall positive experiences, patients identified several challenges related to care delivery. The most frequently cited challenge was staff turnover, which was linked to disrupted continuity and the need to reestablish rapport with each new ICT.

‘… since I’ve been going to the podiatrist, the [ICT] has changed on me about four times. Each time because they are leaving or getting a better job or whatever it is. I go to my appointment and I have new [ICTs]. […] The worst part for me of seeing my podiatrist at VA is these [ICTs] change. Now, you’ve got to try to have a dialogue with a new provider. Sometimes, they don't wanna talk.’ (Patient 14)

Another complaint related to changing ICTs is the variability in care they deliver. It can be challenging to know what level of care to expect when the ICT changes.

‘All technicians are not equal. There are some, I don't know if they’re further along in the program than others, who do an exceptional job. And then there are some who are basically butchers.’ (Patient 6)

Some patients were dissatisfied that the podiatrist was not physically present during their HELPP appointments.

‘I think probably the least desirable [aspect of care] is not having the doctor right there […] So, I would say that's the—I guess the video is probably the low point.’ (Patient 7)

Patients also highlighted challenges with scheduling, particularly when establishing their care.

‘Well, when I first started, and of course they don't know me from Adam, it took a little time to go through the processing. But now that that's all established it's just bang, bang, bang.’ (Patient 16)

Notably, six patients representing the sites labeled VAMC, CBOC 2, and CBOC 3 did not identify any challenges with their HELPP care.

### Suggestions for improvement

Based on patients’ reflections on benefits and challenges with their HELPP care, patients were asked to share their suggestions for improvement.

Patients identified two suggestions to expand HELPP services and connect more patients to HELPP care. First, patients recognized the need to hire more podiatrists and ICTs to ensure HELPP care could be offered to all Veterans who would benefit and enable HELPP to offer appointments on additional days and at more locations to better serve those receiving care.

‘I wish they had the staffing to spread [HELPP] to more people. I wish they had whatever they need to do, because […] I been seein’ people havin’ trouble walkin’ for years […] they just don't know that there is help.’ (Patient 3)

Second, patients advised advertising HELPP more widely to connect more patients to care.

‘They need to let the Veterans know that they’re there. It's like a place putting up an ‘Under New Management’ sign, you know what I mean? People don't like the old management that was there, so they walk right by the place. But when you put up that new management sign, you’re gonna get some more people to walk in there.’ (Patient 21)

Additionally, patients desired improvements to the follow-up care that HELPP offers, including connections to specialty care and consults for shoes.

‘When the doctors gives orders, to me, if the doctor gives orders, it's something that should be followed. Just like I said, it's been more than six months, almost seven or eight months since they ordered the shoes, and I have heard nothing. So, somewhere along the line, the communication is breaking down.’ (Patient 2)

Finally, patients who expressed challenges with the podiatrist not being physically present, suggested that having an in-person podiatrist would be an improvement to the care they receive, though one patient also acknowledged it may not be feasible.

‘The only thing I can think of is [if] the doctor was physically on site, which I know because podiatry is such specialized medicine it's probably hard to do. […] But to have the podiatrist physically present, I think, might go a long way in at least making the Veteran feel more accommodated […] Somebody in-person kinda does give you a little oomph.’ (Patient 5)

There were no suggestions related to technology or scheduling, as technology was not a challenge patients noted and scheduling often resolved after initial hiccups.

## Discussion

Based on a review of podiatry telehealth literature, few, if any, studies have assessed the benefits, challenges, and opportunities for improvement among patients receiving podiatry services via telehealth. Patient reflections revealed high satisfaction with their HELPP care, particularly with the staff providing the care, and several perceived benefits, including improved access, convenience, and foot health. Patients also identified important challenges, such as ICT turnover, the podiatrist being remote, and scheduling issues when establishing care. The identified opportunities for improvement related to hiring more staff, advertising HELPP to further expand access, care coordination (i.e., referrals to other specialties, improved follow-up on shoe orders), and an in-person podiatrist. These evaluation findings have programmatic implications for HELPP and broader implications for hybrid telehealth. Findings have been shared with HELPP leadership and the operational offices supporting this work.

Consistent with prior literature which has found high patient satisfaction with telemedicine across medical specialties, we found HELPP patients were highly satisfied with the podiatry telehealth services they received and felt the services met their needs (13). Similar to benefits reported in other telehealth studies, HELPP patients reported that telehealth resulted in reduced travel burden, improved access, and adequate care (13–16). Notably, HELPP patients brought up staff quality as a benefit of their HELPP care. Few studies have focused on patient assessment of telehealth provider interpersonal skills, though one study found high patient satisfaction with teleradiology providers’ interpersonal skills (17). Additional investigation to identify any commonalities among telehealth services with which patients report high satisfaction could help make determinations of which telehealth models to offer in various circumstance.

Contradictory to other VA telehealth offerings that experienced challenges with technical aspects, internet access, and digital literacy (3, 18), HELPP patients did not bring up technical issues. This may be due to HELPP technology challenges being more visible to the podiatrist or ICT delivering care since the patients do not interact directly with the technology per the HELPP care model. Hybrid telehealth models, like HELPP, may also be advantageous in other clinical settings compared to more conventional telehealth, as limiting the technology burden on patients may allow greater reach to those who would otherwise not engage in telehealth.

Additionally, prior studies found significant challenges with the doctor-patient relationship and note that video visits feel less personal than in-person visits (3, 15). This could account for some patients’ frustration with the podiatrist being remote; however, the addition of an in-person ICT may have lessened this challenge for other HELPP patients. Future work exploring hybrid telehealth models should focus on how to increase visibility of remote providers and the role and importance of an in-person extender, like the HELPP ICT.

The most predominant challenge mentioned by HELPP patients was staff turnover. This aligns with findings that U.S. federal healthcare and behavioral health worker turnover increased in 2024-2025 (19) ICT turnover may also be due to low salaries, which is linked to turnover intention or actual turnover of health technicians working in pharmacy and dialysis settings, respectively (20, 21). Related to HELPP staff turnover, HELPP patients recommended hiring additional ICTs and podiatrists as an improvement opportunity. When patient comments focus on staffing levels and turnover, it suggests that staffing issues are visible and may impact perceived quality of care even when patients assess the staff to be thorough and competent. This aligns with previous studies indicating staff turnover is linked to patient satisfaction and quality of care (22, 23). These findings suggest that telehealth implementation efforts must prioritize staffing stability, not just workforce expansion.

This evaluation had several limitations. First, participants were drawn from a single VA healthcare system, which may limit transferability. Second, only patients who engaged with HELPP were included, and perspectives of those who declined or were unable to access care were not captured. Third, this evaluation relied entirely on patient self-report without triangulation from clinical records, ICT perspectives, and podiatrist perspectives. As such, patient reported improved foot health has not been verified clinically. Fourth, demographic data were not collected, precluding subgroup analyses and potentially limiting the transferability of the findings to other Veteran populations. Finally, interviews occurred during a period of federal system uncertainty, which may have influenced perceptions of VA care.

As telemedicine continues to expand, hybrid workforce models such as HELPP offer a promising approach to extending specialty care. These findings suggest that successful implementation depends not only on access, but also ensuring staffing stability, consistency, and effective communication processes. Future work should include perspectives from ICTs and podiatrists to better understand implementation barriers and facilitators, particularly those related to staffing, and to inform sustainable scale-up of similar models.

## Data Availability

The datasets presented in this article are not readily available because the data were generated as part of a non-research quality improvement evaluation conducted within the United States Department of Veterans Affairs. You may contact the corresponding author to discuss potential availability of specific data elements. Requests to access the datasets should be directed to Lacey Evans, Lacey.Evans@va.gov.
