# Supplementary material for: Patient reflections on benefits, challenges, and improvement opportunities for a VA podiatry telehealth program: a qualitative study
Source: Front Digit Health. 2026 Aug 3;8:1904512. doi: 10.3389/fdgth.2026.1904512 (PMC13478303; doi:10.3389/fdgth.2026.1904512)
Supplement: Supplementary file 1 [file Table1.docx]

**Supplementary File 1: HELPP Interview Guide**

1.	Tell me a little about your experience receiving podiatry (foot care) services.

2.	Were you receiving podiatry services in the community, or through community care before coming to VA?

a.	If so, did you have any concerns about transferring services to VA?

b.	How did you hear about podiatry services at VA (e.g., PCP)?

c.	Why did you choose to transfer to VA podiatry?

d.	Were you receiving podiatry services at another VA location in the Atlanta healthcare system, such as another community-based outpatient clinic (CBOC)?

Now we’re going to focus on your experiences receiving podiatry services within VA.

3.	What made you interested in participating in VA podiatry services?

a.	How long had you been experiencing issues prior to receiving VA podiatry services?

b.	Were there particular podiatry concerns that you wanted to have addressed?

c.	How long have you been receiving podiatry services at VA?

d.	Where do you primarily receive your podiatry care? VA medical center, outpatient clinic, or both?

4.	Tell me what it’s like receiving podiatry services at VA. Walk me through your typical appointment.

a.	What challenges, if any, have you encountered when scheduling podiatry appointments at VA?

b.	Tell me a little bit about the staff you interact with during these appointments.

c.	Are there certain staff you feel most comfortable with?

d.	Have you been offered appropriate footwear, such as diabetic shoes, during your VA podiatry appointment?

i.	If yes, have you received this footwear?

ii.	Have you experienced any trouble receiving this footwear?

5.	Would you describe the podiatry services you are receiving as virtual care? Or in-person care?

6.	What has been the best part of receiving your podiatry services at VA (e.g., wait times, drive times, greater availability of providers/services, convenience)?

7.	What has been the worst part of receiving your podiatry services at VA (e.g., less rapport with the doctor, communication challenges, equipment, technology, ability to hear, ability to see)?

8.	Do you have any foot concerns that you were expecting to have addressed by your podiatry providers, that you did not? If yes, what are they

a.	How have you communicated these concerns to your provider?

b.	What kinds of actions would you like your provider to take to address these concerns?

9.	How can we reach more Veterans to engage with podiatry services at VA?

a.	Are there any types of Veterans that you feel we should be targeting more to engage with these services (e.g., women, rural, elderly, etc.)?

b.	Why do you think these types of Veterans would benefit from these services?

10.	Has your experience with podiatry services at VA had any influence on your decision to seek out other VA care?

11.	Do you have any suggestions to improve the way VA podiatry services are delivered?

a.	Some people find it beneficial to combine health screenings in one appointment. Would you find it valuable to add eye screenings to your podiatry appointments? Why or why not?

12.	Would you want to continue to receive VA podiatry services in this way (i.e., virtually) going forward?

a.	If yes, what kinds of resources would you need to ensure continued participation in services?

b.	If no, what would make you want to use this service in the future?

13.	Is there anything else that we have not covered that you think would be important for us to know regarding podiatry services and overall footcare at VA?
